# A statistical framework for integrating two microarray data sets in differential expression analysis

**DOI:** 10.1186/1471-2105-10-S1-S23

**Published:** 2009-01-30

**Authors:** Yinglei Lai, Sarah E Eckenrode, Jin-Xiong She

**Affiliations:** 1 Department of Statistics and Biostatistics Center, The George Washington University, 2140 Pennsylvania Avenue, N.W., Washington, D.C. 20052, USA; 2 Center for Biotechnology and Genomic Medicine, Medical College of Georgia, 1120 15th street, CA4098, GA 30912, USA

## Abstract

**Background:**

Different microarray data sets can be collected for studying the same or similar diseases. We expect to achieve a more efficient analysis of differential expression if an efficient statistical method can be developed for integrating different microarray data sets. Although many statistical methods have been proposed for data integration, the genome-wide concordance of different data sets has not been well considered in the analysis.

**Results:**

Before considering data integration, it is necessary to evaluate the genome-wide concordance so that misleading results can be avoided. Based on the test results, different subsequent actions are suggested. The evaluation of genome-wide concordance and the data integration can be achieved based on the normal distribution based mixture models.

**Conclusion:**

The results from our simulation study suggest that misleading results can be generated if the genome-wide concordance issue is not appropriately considered. Our method provides a rigorous parametric solution. The results also show that our method is robust to certain model misspecification and is practically useful for the integrative analysis of differential expression.

## Background

Microarray is an experimental method by which tens of thousands of genes can be printed on a small chip and their expression can be measured simultaneously [1,2]. Since the microarray technology was introduced, it has been widely used in many biomedical studies [3,4]. Microarrays can be used to measure expression for tens of thousands of genes at the mRNA level for samples in normal and disease groups, and then statistical methods for two-sample comparison can be used to identify differentially expressed genes. Differentially expressed genes are potential disease related genes for clinical diagnoses and medical treatments. This approach has been successfully used in cancer studies [4,5] as well as diabetes studies [6,7].

Although microarray technology has been developed for more than a decade, the experiment cost is still considerably expensive. This limits the sample size of microarray studies. Therefore, the detection power can be low, especially when the signal of differential expression is relatively weak [8]. Many microarray data sets have been collected for the same or similar research purpose. Detecting genes with concordant behavior among different data sets is of biological interest. It is also of statistical interest to improve the detection power if it is feasible to integrate different data sets in differential expression analysis. For this reason, several methods have been proposed for data integration [9-14].

However, the genome-wide concordance of different data sets has not been well considered in these integrative analyses. A gene selected for the follow-up analysis should behave concordantly in different data sets. For example, if a gene is up-regulated in one experiment, then it should also be up-regulated in another experiment. Slight inconsistency should be expected since there are considerable noises generated by microarray experiments. If two data sets are genome-wide concordant, then integrating them can generally improve the sample size and reduce the noise impact. Therefore, it is desirable to combine observations of concordant genes since we expect to achieve a more powerful detection of differential expression. However, if two data sets are not genome-wide concordant, then there are genes with discordant behavior in different data sets. There are many possible factors for such observations, such as population heterogeneity, probe binding issues from different microarray platforms, as well as lab-specific system noises. Therefore, integrating observations of discordant genes may result in misleading conclusions and should be discouraged.

When a seemingly discordant behavior is observed for a gene, it is difficult to tell whether the observation is generated by random noises or the observation reflects the underlying truth. Therefore, it is not trivial to determine whether a gene has a concordant/discordant behavior in different experiments. The analysis will be more complicated for evaluating genome-wide concordance. Cahan et al. [15] have studied different gene lists identified from different data sets. Ein-Dor et al. [16] have showed that we may need to collect thousands of samples to generate a robust gene list for disease prediction. Miron et al. [17] have proposed a correlation based approach for measuring concordance between two lists of test statistics from two data sets. However, this approach does not consider the fact that different genes in a data set belong to different components (non-differentially expressed, up-regulated, down-regulated, etc.). We have recently proposed a mixture-model based method for testing genome-wide concordance and discordance [18]. This approach considers the mixture of different gene components as well as the independence between two data sets, and it can be extended for data integration. In this study, we propose a mixture-model based statistical framework to achieve a rigorous integrative analysis of differential expression.

In a recent study [19], it has been shown that the widely used overlap count (or Venn diagrams) is not an appropriate metric for measuring the reproducibility of differential expression analysis. It is necessary to develop new metrics for rigorously measuring the reproducibility of differential expression analysis. The disadvantage of overlap count metric is that the randomness of differential expression measures (e.g. *t*-test) has not been well considered. However, our mixture-model based tests of genome-wide concordance and discordance [18] take this randomness into account and the reported *p*-values can be used as rigorous metrics for measuring the reproducibility of differential expression analysis.

For the rest of paper, we first introduce our statistical framework. Then, we use simulated data to evaluate its performance. Two experimental data based case studies are considered as the applications. Finally, we discuss the advantages and disadvantages of our method.

## Methods

### A statistical framework

**Figure 1:**
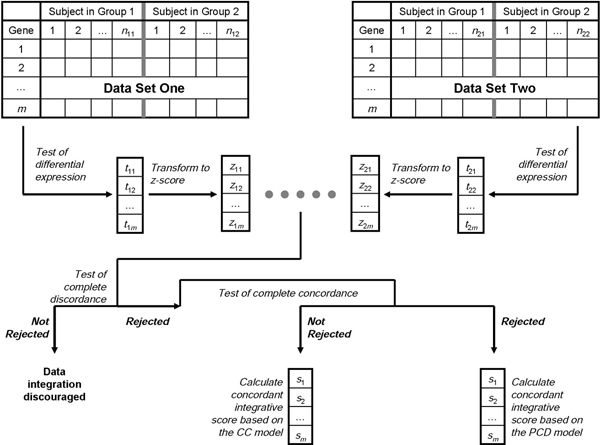
**Flow chart**. A flow chart illustrates our statistical framework. The details are provided in the Methods section.

Figure 1 provides an illustrative flow chat for our statistical framework. The integration of two microarray gene expression data sets is considered so that we can achieve a more powerful detection of concordantly differentially expressed genes. Here, we assume that two data sets have been pre-processed so that they contain the same gene list. The framework can be summarized as the procedure below. Then, we describe the detail of each step. (See our recent publication [18] for the technical detail of tests of complete concordance and complete discordance.)

1. In each data set, perform a statistical test of differential expression for each gene to obtain a lists of test scores;

2. For each list of test scores, perform a transformation procedure to obtain a list of *z*-scores;

3. For two lists of *z*-scores, test the complete discordance between them;

(a) if the complete discordance cannot be rejected, then the data integration will be discouraged;

(b) if the complete discordance can be rejected, then continue to the next step;

4. For two lists of *z*-scores, test the complete concordance between them;

(a) if the complete concordance cannot be rejected, then calculate a list of concordant integrative scores based on the complete concordance (CC) model;

(b) if the complete concordance can be rejected, then calculate a list of concordant integrative scores based on the partial concordance/discordance (PCD) model;

5. Use the list of concordant integrative scores to prioritize genes for the follow-up study.

### Test of differential expression

For simplicity, we consider the Student's two-sample *t*-test for differential expression analysis. Other test statistics, such as Wilcoxon's rank sum test or a generalized *t*/*F*-statistic [20], can certainly be considered. The statistical significance (*p*-value) of a test value can be evaluated based on either its theoretical null distribution or a permutation null distribution [21]. In this study, the theoretical *p*-value is used for the simulation study since we know the underlying distribution; the permutation *p*-value is used for the application since the underlying distribution is unknown (*B *= 500 is used as the number of permutations).

### Transformation of test score

It has been suggested transforming a test value to its associated *z*-score so that more efficient results can be achieved in a normal mixture model based analysis [22]. When the one-sided (upper-tailed) *p*-value of a test value is available, the associated *z*-score can be simply calculated by

*z *= Φ^-1^(1 - *p*),

where Φ^-1^(·) is the inverse of standard normal distribution. Notice that it is necessary to use one-sided *p*-values since we intend to distinguish up-regulated differential expression from down-regulated differential expression.

### Mixture models

We have proposed several mixture models [18] to evaluate the genome-wide concordance/discordance between two lists of *z*-scores: {(*z*_1*k*_, *z*_2*k*_) : *k *= 1, 2, ..., *m*}, where *m *is the number of common genes in both data sets. A general mixture model can be used to represent the case of partial concordance/discordance (PCD):

$$f_{\text{PCD}}(z_{1},z_{2})=\sum_{i=0}^{2}\sum_{j=0}^{2}\pi_{ij}\phi_{\mu_{i},\sigma_{i}^{2}}(z_{1})\phi_{\nu_{j},\tau_{j}^{2}}(z_{2}).$$

This model can be reduced to a complete concordance (CC) model:

$$f_{\text{CC}}(z_{1},z_{2})=\sum_{i=0}^{2}\pi_{ii}\phi_{\mu_{i},\sigma_{i}^{2}}(z_{1})\phi_{\nu_{i},\tau_{i}^{2}}(z_{2}),$$

and a complete discordance (CD) model:

$$f_{\text{CD}}(z_{1},z_{2})=[\sum_{i=0}^{2}\pi_{i}.\phi_{\mu_{i},\sigma_{i}^{2}}(z_{1})][\sum_{j=0}^{2}\pi ._{j}\phi_{\nu_{j},\tau_{j}^{2}}(z_{2})].$$

More details for these models have been described in our recent publication [18]. In these models, index 0 is used to represent the null component with fixed parameters: *μ*_0 _= *ν*_0 _= 0 and $\sigma_{0}^{2}=\tau_{0}^{2}=1$; indices 1 and 2 are used to represent the down-regulated and up-regulated components with constrains: *μ*_1_, *ν*_1 _≤ 0 and *μ*_2_, *ν*_2 _≥ 0; *π*_*ij *_is the proportion of genes belonging to the *i*-th component in the first data set and *j*-th component in the second data set (Σ_*ij *_*π*_*ij *_= 1). *π*_*i*. _is the marginal proportion of genes belonging to the *i*-th component in the first data set; and *π*._*j *_is the marginal proportion of genes belonging to the *j*-th component in the second data set. The model parameters can be estimated through an E-M algorithm [23]. The detail has also been described in our recent publication [18].

### Tests of concordance and discordance

Based on the assumption of independence among the list of *z*-scores, we can calculate the mixture model based likelihoods:

$$L_{\text{PCD}}=\prod_{k=1}^{m}f_{\text{PCD}}(z_{1k},z_{2k});$$

$$L_{\text{CC}}=\prod_{k=1}^{m}f_{\text{CC}}(z_{1k},z_{2k});$$

$$L_{\text{CD}}=\prod_{k=1}^{m}f_{\text{CD}}(z_{1k},z_{2k}).$$

With these likelihoods, we can test PCD (*H*_1_) against CC (*H*_0_) or CD (*H*_0_) by the following likelihood ratio tests in the logarithm scale:

$$\begin{array}{c} T_{\text{CC}}=\log(L_{\text{PCD}}/L_{\text{CC}})=\log(L_{\text{PCD}})-\log(L_{\text{CC}});\\ T_{\text{CD}}=\log(L_{\text{PCD}}/L_{\text{CD}})=\log(L_{\text{PCD}})-\log(L_{\text{CD}}). \end{array}$$

The statistical significance of a test value can be evaluated by the parametric bootstrap procedure [24], which has also been described in our recent publication [18].

### Data integration

If the complete discordance (CD) cannot be rejected, then the data integration will be discouraged to avoid misleading results. If CD can be rejected, then either the complete concordance (CC) or the partial concordance/discordance (PCD) will be established. Although CC is a special case of PCD, it is still statistically necessary to test PCD against CC. If CC cannot be rejected, then we expect to achieve a more efficient data integration by reducing the number of parameters. Under CC or PCD, it is feasible to consider the data integration. To prioritizing genes, we can consider a concordant integrative score, which is the conditional probability of concordantly differential expression under an appropriate mixture model: [P(observed pair of *z*-scores both up-regulated) + P(observed pair of *z*-scores both down-regulated)]/P(observed pair of *z*-scores). Under the CC model, it is calculated as:

$$S_{\text{CC}}(z_{1},z_{2})=\frac{\sum_{i=1}^{2}{\hat{\pi }}_{ii}\phi_{{\hat{\mu }}_{i},{\hat{\sigma }}_{i}^{2}}(z_{1})\phi_{{\hat{\nu }}_{i},{\hat{\tau }}_{i}^{2}}(z_{2})}{\sum_{i=0}^{2}{\hat{\pi }}_{ii}\phi_{{\hat{\mu }}_{i},{\hat{\sigma }}_{i}^{2}}(z_{1})\phi_{{\hat{\nu }}_{i},{\hat{\tau }}_{i}^{2}}(z_{2})};$$

Under the PCD model, it is calculated as:

$$S_{\text{PCD}}(z_{1},z_{2})=\frac{\sum_{i=1}^{2}{\hat{\pi }}_{ii}\phi_{{\hat{\mu }}_{i},{\hat{\sigma }}_{i}^{2}}(z_{1})\phi_{{\hat{\nu }}_{i},{\hat{\tau }}_{i}^{2}}(z_{2})}{\sum_{i=0}^{2}\sum_{j=0}^{2}{\hat{\pi }}_{ij}\phi_{{\hat{\mu }}_{i},{\hat{\sigma }}_{i}^{2}}(z_{1})\phi_{{\hat{\nu }}_{j},{\hat{\tau }}_{j}^{2}}(z_{2})}.$$

### False positive control

The mixture model provides a rigorous convenience to estimate the number of false positives in a theoretical manner. Notice that the above concordant integrative score is actually a probability of true positive. Therefore, if we are interested in the top *K *genes {*X*_(1)_, *X*_(2)_, ..., *X*_(*K*)_} ranked by the concordant integrative score, then the associated number of false positives can be estimated as:

$$\hat{FP}=K-\sum_{k=1}^{K}S(z_{1,(k)},z_{2,(k)}),$$

where *S*(·) is calculated based on an appropriate mixture model (CC or PCD). With this estimate, one may realize that the false discovery rate [25] (FDR) based on the *q*-value concept [26] can be simply estimated as $\hat{FP}$/*K*.

For an individual data set, we simply use the R-package **qvalue **[26] to obtain the estimated FDR. Then, the number of false positives for the top *K *genes can be simple estimated as *K *× *FDR*(*K*), which is theoretically consistent with the above $\hat{FP}$.

## Results and discussion

### Illustrative examples

**Figure 2:**
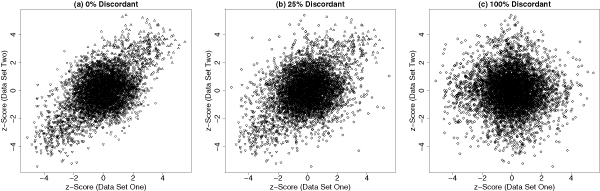
I**llustrative examples**. Three examples demonstrate the concepts of genome-wide concordance and discordance. The up-triangles, down-triangles and circles represent the concordantly up-regulated, down-regulated and null genes; the diamonds represent the discordantly expressed genes.

Figure 2 shows three examples to illustrate the concepts of concordance and discordance. These examples are simulated based on the simulation configuration in the next subsection. The proportions of genes with discordant behavior in two data sets are *ξ *= 0%, 50% and 100%, respectively. Therefore, these are representative examples for the cases of complete concordance, partial concordance/discordance and complete discordance. The corresponding Pearson's correlation coefficients are 49.2%, 36.1% and -2.1%. Therefore, the correlation measure is not an appropriate metric to tell whether two data sets are concordant or not. One may also realize that the overlap count is neither a rigorous approach even for the first example (a case of complete concordance).

### A simulation study

There are many parameters to be considered when we simulate microarray gene expression data:

• Gene size *m*;

• The proportion of non-differentially expressed genes *π*_0_;

• Sample sizes of two groups *n*_1_, *n*_2_;

• Distributions of expression measurements of differentially and non-differentially expressed genes;

• Covariance structure among genes.

In our simulation studies, we consider the widely used block structure: genes are partitioned into many blocks; genes within the same block are positively dependent; and different blocks are independent. To save the computing time, we reasonably set gene size *m *= 6000, *π*_0 _= 80% and *n*_1 _= *n*_2 _= 15 for each of two data sets. The block size (number of genes in each block) is set *b *= 25. Within each block, the expression measurements are simulated from a multivariate normal distribution. For blocks of non-differentially expressed genes, we simulate expression measurements from *N*(${\hat{\mu }}_{0}$, Σ_0_), where ${\hat{\mu }}_{0}$ is a *b *× 1 vector of 0's and Σ_0 _is a *b *× *b *matrix with diagonal entries as 1 and non-diagonal entries as a fixed value (simulated from a Uniform distribution *U*[0.5, 0.9]). For blocks of differentially expressed genes, we simulate expression measurements from *N*(${\hat{\mu }}_{1}$, Σ_1_) and *N*(${\hat{\mu }}_{2}$, Σ_2_) for the first and the second sample groups, respectively. ${\hat{\mu }}_{2}$ is simply a *b *× 1 vector of 0's. To simulate ${\hat{\mu }}_{1}$, we first simulate *b *× 1 vector of random numbers from a Beta distribution *Beta*(1.5, 1.5), multiply this vector by a factor *r *= 1.5, and then multiply randomly simulated signs (50% positive and 50% negative) so that both up and down regulated differential expression can be generated. Σ_1 _and Σ_2 _are similarly generated as Σ_0_. Two data sets are first simulated based on the same configuration. To simulate genes with discordant behavior, we randomly reallocate *ξ *= 0%, 15%, 30%, 45%, 60% and 75% genes in the second data set so that these genes are no longer matched with those in the first data set. Notice that the simulation configuration is not completely consistent with what have been assumed for our method. This is intentionally designed to understand the robustness of our method. The complete concordance or the complete discordant will be rejected at the level *p*-value < 0.025 since there is a issue of multiple hypothesis testing. To save computing time, we only perform the parametric bootstrap for 100 times to evaluate the *p*-value of a test.

For each round of simulation, since we know the truth (simulation configuration), we can use the curve of number of concordantly differentially expressed genes (True Positives) against number of claimed ones (Claimed Positives) to evaluate the performance of our method. After many (50) rounds of simulations (it takes a long time for each round due to the parametric bootstrap procedure with the E-M algorithm based estimation), we can take the average to obtain a smooth mean curve. Since the existing data integration methods [9-14] do not consider the issue of genome-wide concordance/discordance, they are not included for comparison in this study. However, we have compared our method with the simple pooling approach: observations of the same gene from two data sets are simply combined for each sample group, and the *t*-test is applied to each gene in the pooled data set. This approach is feasible since the measurements in two simulated data sets are comparable. (Then, this approach is a desired efficient approach when two data sets are genome-wide concordant. It is interesting to understand its loss of power when two sets are not genome-wide concordant.)

**Figure 3:**
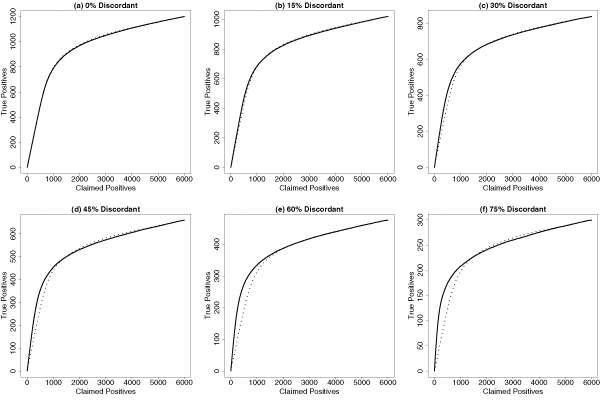
**Simulation results**. The curves of number of truly concordantly differentially expressed genes (True Positives) against number of claimed ones (Claimed Positives) are used to evaluate the performance of our method. Different proportions of discordant genes (0 ~ 75%) are considered. The solid curves represent our mixture-model based approach; the dotted curves represent the pooling approach for a comparison.

Figure 3 shows the comparison between our method and the pooling approach. When *ξ *= 0% (complete concordance), the performance of two approaches are still comparable. (Notice that in such a situation, the pooling approach is an ideal choice.) Our tests of complete concordance (CC) and complete discordance (CD) result in 45 CC and 5 partial concordance/discordance (PCD) among 50 repetitions. The advantage of our method becomes clearer and clearer when *ξ *is increased from 15% to 75% (partial concordance/discordance): our method is clearly better when the number of claimed positives is within 1 to 1000 (notice that relatively lowly ranked genes are of less interest in microarray studies). Our tests of CC and CD result in 50 PCD among 50 repetitions for all these 5 configurations. In addition to the practical usefulness of our method, Figure 3 also confirms that it is importance to evaluate the genome-wide concordance before the data integration can be considered. Otherwise, we may obtain seriously misleading analysis results.

### Applications

#### A case study of partial concordance/discordance

Since NOD mouse spontaneously develops type 1 diabetes, it has been widely used for studying the disease. Based on a time course microarray study using samples collected from 3 weeks to 10 weeks, week 5 is a key checkpoint for the development of type 1 diabetes [7]. To distinguish the genes related to diabetes development from the genes related to aging, two other data sets have also been collected for two congenic strains: NOD.Idd3/Idd10 and NOD.B10Sn-H2, which do not spontaneously develop diabetes. Samples have been collected at different time points from 3 weeks to 10 weeks [7]. Although these two strains do not spontaneously develop type 1 diabetes, it is still interesting to understand their differential expression before 5 weeks vs. after 5 weeks. Furthermore, understanding genes with concordant/discordant behavior for these two strains is important. Therefore, the data set collected for each congenic strain is partitioned into two sample groups: for strain NOD.Idd3/Idd10, there are 11 and 13 subjects collected before 5 weeks and after 5 weeks, respectively; for strain NOD.B10Sn-H2, there are 22 and 10 subjects collected before 5 weeks and after 5 weeks, respectively. Measurements for 11,424 genes have been collected based on a cDNA microarray platform.

**Figure 4:**
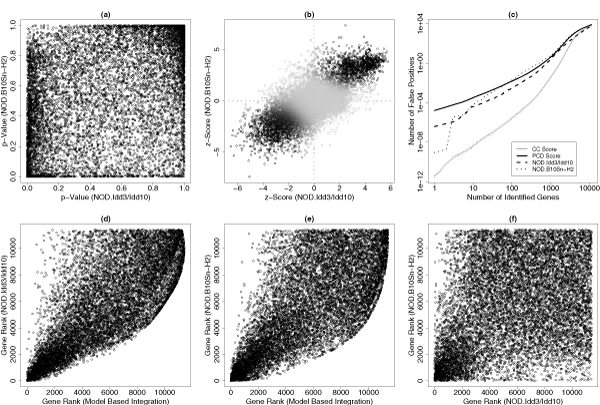
**Application results**. Two scatter-plots show the paired *p*-values (a) and the paired *z*-scores (b) from two NOD mouse data sets. The false discovery rate curves (c) compare the results based on the PCD model based data integration, the CC model based data integration and two individual sets. The gene ranks based on the PCD model based integration and two individual data sets are also compared (d-f).

Figures 4a and 4b show the scatter-plot for the paired *p*-values (based on 500 permutations) and *z*-scores of 11,424 genes based on these two data sets. It is difficult to evaluate the genome-wide concordance/discordance based on the scatter-plot of paired *p*-values (Figure 4a). From the scatter-plot of paired *z*-scores (Figure 4b), the genome-wide concordance seems quite satisfactory. However, based on 1000 parametric bootstraps (Table 1), the tests of complete concordance (CC) and complete discordance (CD) are both significant (*p *< 0.01). Therefore, both CC and CD models are rejected and the partial concordance model (PCD) should be used in the analysis. This is not surprising since certain genetic and biological differences are expected from these two similar strains. Table 2 gives the PCD model estimates. There are still about 75% genes with concordant behavior. The level of darkness in Figure 4b represents the level of being concordantly differentially expressed that is evaluated by the concordant integrative score based on the PCD model (we set 0.2 for the smallest darkness level so that these non-differentially expressed genes can be visualized).

**Table 1:** **Application results.** The parametric bootstrap based null quantiles are used to evaluate the significance (*p*-values) of the tests of complete discordance and complete concordance between two NOD mouse data sets.

		Quantile under Null				
Test	Observed	90%	95%	99%	99.5%	99.9%

Complete Discordance (*T*_CD_)	2477.1	2.6	3.2	4.6	5.5	7.3
Complete Concordance (*T*_CC_)	-269.3	-378.9	-371.6	-350.9	-345.8	-340.5

**Table 2:** **Application results. **The parameters in the PCD model for two NOD mouse data sets are estimated through an E-M algorithm.

		Data Set Two (NOD.B10Sn-H2)				
		Down	Null	Up	Mean	Variance
Data Set One	Down	0.150	0.078	0.001	-2.424	1.279
	Null	0.076	0.488	0.018	0	1
(NOD.Idd3/Idd10)	Up	0.002	0.079	0.109	2.685	1.493

Mean		-2.032	0	3.209		
Variance		1.333	1	0.777		

Figure 4c shows the estimated false discovery rates. Compared to the analysis based on individual data sets, the PCD model based false positive control is not necessarily better since we intend to detect these concordantly differentially expressed genes in the integrative data analysis. Furthermore, it is important that an appropriate model must be used for the data integration. Figure 4c also shows that the CC model based analysis results can be seriously misleading. Therefore, the tests of complete concordance and complete discordance are crucial before the data integration can be considered. Figure 4d–f compare the gene ranks based on the PCD model based integration and two individual data sets. The gene ranks based on two individual data sets are quite discordant: the Spearman's rank correlation is just 0.36. The integration based gene ranks and these based on two individual data sets are quite concordant: the Spearman's rank correlations are 0.78 and 0.72, respectively.

#### A case study of complete concordance

In practice, we may collect gene expression data for the same study from different laboratories based on different microarray platforms. These data usually cannot be directly combined for an analysis with a larger sample size. Our method can also be used to solve this problem. Although this situation has been discussed in our simulation study, it is still necessary to illustrate it with experimental data. Here, we generate a case of complete concordance based on an experimental data set. The data set was collected for a prostate cancer study [5]. Genome-wide expression profiles for 6034 genes (after data preprocessing) have been measured for 50 normal and 52 cancerous subjects. We randomly split this data set into two subsets with equal sample sizes (25 normal and 26 cancerous subjects).

**Figure 5:**
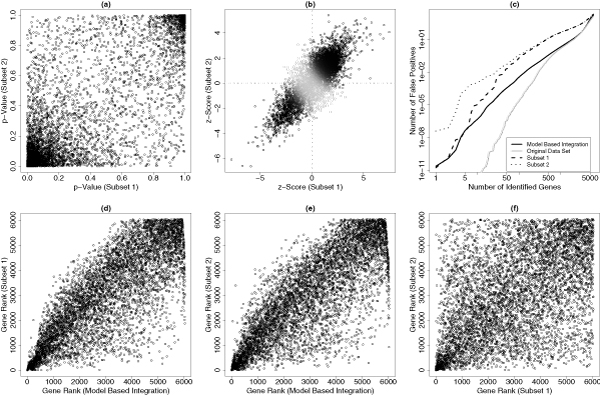
**Application results**. Two scatter-plots show the paired *p*-values (a) and the paired *z*-scores (b) from two prostate cancer data subsets. The false discovery rate curves (c) compare the results based on the CC model based data integration, the original data (subsets 1 and 2 pooled together) and two individual subsets. The gene ranks based on the CC model based integration and two individual subsets are also compared (d-f).

Figures 5a and 5b show the scatter-plot for the paired *p*-values (based on 500 permutations) and *z*-scores of 6,034 genes based on these two subsets. They are highly genome-wide concordant. This is consistent with our expectation. Based on 1000 parametric bootstraps (Table 3), the complete discordance is rejected (*p *< 0.01) but the complete concordance cannot be rejected (the associated *p*-value is highly insignificant). Table 4 gives the CC model estimates. The estimates of mean and variance parameters in two subsets are consistent. The level of darkness in Figure 5b represents the level of being concordantly differentially expressed that is evaluated by the concordant integrative score based on the CC model (0.2 is set for the smallest darkness level so that these non-differentially expressed genes can be visualized).

**Table 3:** **Application results.** The parametric bootstrap based null quantiles are used to evaluate the significance (*p*-values) of the tests of complete discordance and complete concordance between two NOD mouse data sets.

		Quantile under Null				
Test	Observed	90%	95%	99%	99.5%	99.9%
Complete Discordance (*T*_CD_)	1515.8	1.1	1.4	2.4	2.6	3.2
Complete Concordance (*T*_CC_)	-635.4	-264.4	-257.2	-245.7	-241.8	-234.9

**Table 4:** **Application results.** The parameters in the CC model for two prostate cancer subsets are estimated through an E-M algorithm.

		Subset 2				
		Down	Null	Up	Mean	Variance
Subset 1	Down	0.157	-	-	-2.290	0.987
	Null	-	0.273	-	0	1
	Up	-	-	0.570	1.685	0.629

Mean		-2.123	0	1.678		
Variance		0.924	1	0.547		

Figure 5c shows the estimated false discovery rates. Compared to the analysis based on individual data sets, the CC model based false positive control shows a clear improvement. However, the false positive control based on the original data (subsets 1 and 2 pooled together) is the best. This is consistent with our simulation results. Figure 5d–f compare the gene ranks based on the CC model based integration and two individual subsets. The gene ranks based on two individual subsets are quite discordant: the Spearman's rank correlation is just 0.50. The integration based gene ranks and these based on two individual data sets are quite concordant: the Spearman's rank correlations are both 0.81. Furthermore, the integration based gene ranks are highly concordant with these based on the original data (result not shown): the Spearman's rank correlation is 0.96.

## Conclusion

In this study, we have proposed a statistical framework for integrating two microarray gene expression data sets in differential expression analysis. Our simulation and application results confirm that it is necessary to evaluate the genome-wide concordance before the consideration of data integration. Otherwise, misleading results can be generated from the integrative analysis. Our current study focuses on the integration of two data sets with two-sample groups. In our future study, we will generalize our method for multiple data sets. However, it is less straightforward to generalize our method for multi-sample groups since it is difficult to define the concordance/discordance for multiple groups.

Because of the randomness of data, we can always observe some intersection of genes selected from two data sets if the selection criterion is not stringent. This is the case even when two data sets are completed unrelated. (If the selection criterion is stringent, then we may always observe a null intersection even when two data sets are actually related.) Therefore, the genome-wide concordance/discordance is a critical issue in the integrative analysis microarray data. The traditional hyper-geometric analysis relies on the criterion of gene selection, which can be quite arbitrary in practice. For example, the results based on the threshold of 5%, 10% or 20% false discovery rates can be considerably different. It is not a rigorous approach to address the genome-wide concordance/discordance. In a recent study [19], it has also been shown that the widely used overlap count (or Venn diagrams) is not an appropriate metric for measuring the reproducibility of differential expression analysis. Furthermore, it is not clear how to rank genes efficiently in the intersection of genes selected from two data sets.

To our knowledge, there is no other existing methods for evaluating genome-wide concordance/discordance before the consideration of data integration. Our mixture model based approach is simple and intuitive. There are usually 3 major gene groups in a data set: up-regulated, down-regulated and null genes, which correspond to the three components in our model. The model inference is well-developed in the field of statistics. Furthermore, our model allows us to provide rigorous ranks for genes analyzed in two data sets. In our simulation study, our method can still provide a comparable performance in the situation of complete genome-wide concordance when the ideal pooling approach is feasible. If two data sets are not completely concordant, then our method will provide a better performance.

Our method has several advantages. It allows us to test genome-wide concordance/discordance, which is a critical issue before the data integration can be considered. It is a likelihood-based approach, which is efficient when the underlying model is not seriously mis-specified. We have also showed the robustness of our method through a simulation study when the underlying models are somewhat inconsistent. Furthermore, the data integration is achieved through a rigorously defined probability with close formulas.

Our method also has the following disadvantages. It is difficult to validate the assumed mixture model. However, without this assumption, we currently have no effect approach for evaluating genome-wide concordance/discordance. Furthermore, the calculation of likelihood assumes that the test scores from different genes are independent. However, it is well-known that the covariance structure of a microarray gene expression data set can be complicated. In our future study, we will explore more efficient approaches to overcome these disadvantages.

## Competing interests

The authors declare that they have no competing interests.

## Authors' contributions

Y Lai conceived of the study, developed the methods, performed the statistical analysis, and drafted the manuscript; SE Eckenrode carried out the microarray experiments; J-X She designed the microarray experiments and helped to draft the manuscript. All authors read and approved the final manuscript.
